# Evaluating the Process and Extent of Institutionalization: A Case Study of a Rapid Response Unit for Health Policy in Burkina Faso

**DOI:** 10.15171/ijhpm.2017.39

**Published:** 2017-04-10

**Authors:** Andre Zida, John N. Lavis, Nelson K. Sewankambo, Bocar Kouyate, Salimata Ouedraogo

**Affiliations:** ^1^ Clinical Epidemiology and Biostatistics Unit, College of Health Sciences, Makerere University, Kampala, Uganda.; ^2^ Ministry of Health, Ouagadougou, Burkina Faso.; ^3^ McMaster Health Forum, Centre for Health Economics and Policy Analysis, McMaster University, Hamilton, ON, Canada.; ^4^ Department of Health Research Methods, Evidence and Impact, and Department of Political Science, McMaster University, Hamilton, ON, Canada.

**Keywords:** Institutionalization, Rapid Response Service, Burkina Faso, Health Resources, Knowledge Translation

## Abstract

**Background:** Good decision-making requires gathering and using sufficient information. Several knowledge translation platforms have been introduced in Burkina Faso to support evidence-informed decision-making. One of these is the rapid response service for health. This platform aims to provide quick access for policy-makers in Burkina Faso to highquality research evidence about health systems. The purpose of this study is to describe the process and extent of the institutionalization of the rapid response service.

**Methods:** A qualitative case study design was used, drawing on interviews with policy-makers, together with documentary analysis. Previously used institutionalization frameworks were combined to guide the analysis.

**Results:** Burkina Faso’s rapid response service has largely reached the consolidation phase of the institutionalization process but not yet the final phase of maturity. The impetus for the project came from designated project leaders, who convinced policy-makers of the importance of the rapid response service, and obtained resources to run a pilot. During the expansion stage, additional policy-makers at national and sub-national levels began to use the service. Unit staff also tried to improve the way it was delivered, based on lessons learned during the pilot stage. The service has, however, stagnated at the consolidation stage, and not moved into the final phase of maturity.

**Conclusion:** The institutionalization process for the rapid response service in Burkina Faso has been fluid rather than linear, with some areas developing faster than others. The service has reached the consolidation stage, but now requires additional efforts to reach maturity.

## Background

Decision-making is a cognitive process that results in the selection of a course of action from among several alternative scenarios. The decision is either rational or irrational, and based on explicit or tacit assumptions.^1,2^ This applies to all situations, including decision-making in health policy. Good decision-making requires the gathering of sufficient information.^3^ A good decision must not only reflect the preferences of the decision-maker but also be logical and based on the available information.^4^ Evidence from research, particularly high-quality research, is a useful and important source of reliable and up-to-date information in healthcare policy and policy-making more generally.^5^ Several studies have suggested that policy-making informed by evidence is likely to be more effective and less expensive.^6-8^ In practice, however, evidence from research is not always taken into account.

One review,^9^ for example, examined the use of health research in policy-making and its contribution to effective policies. It found that the level of research utilization by policy-makers was lower than it could be. Other reviews have identified factors influencing the use of research in decision-making among health policy-makers.^10,11^ These included interaction, trust and personal contact between researchers and policy-makers, timeliness, the relevance of the research findings, the inclusion of summaries with clear recommendations, and power and budget struggles.

Some countries have therefore taken action at national level to link research to policy-making.^12^ One study looking at this national action used a framework to explain success in increasing the use of evidence. This framework has four components, suggesting that introducing evidence-driven policy-making is a complex process. The first assesses the general climate and how those who fund research, universities, researchers and users of research support or place value on efforts to link research to action. The second addresses the production of research, including how priority-setting ensures the identification of user needs and how scoping analyses, systematic reviews, and simple studies have been undertaken to meet these needs. The third addresses the use of a combination of activities to link research to action.

These include four areas:

(i) Efforts to “push” (or disseminate) research evidence to those who can act on it;
(ii) Efforts to support “user pull,” such as one-stop shops for high-quality reviews, or electronic libraries for health;
(iii) “User pull” itself, efforts undertaken by those who use research; and
(iv) Exchange efforts, or how meaningful partnerships between researchers and users help them to jointly ask and answer relevant questions.

The fourth component addresses approaches to evaluation, including how to support rigorous evaluation of efforts to link research to action.

To improve use of evidence in policy-making, several knowledge translation platforms have been introduced in Burkina Faso to ‘push’ research evidence out to policy-makers, and respond to user ‘pull’ or demand for more information (areas (*i*) and (*ii*) from the third component of the framework described above). One of these is the Supporting the Use of Research Evidence (SURE) project’s health policy rapid response unit, run with European Union (EU) funding. This unit, which draws on a model used in Uganda,^13,14^ sits centrally in Burkina Faso’s health ministry.

This unit runs a service (the rapid response service) to provide health policy-makers in Burkina Faso with rapid access to a summary of relevant research evidence about a particular area of health policy or the health system. This is designed to provide rapid access to detailed briefs of research evidence that address policy makers’ urgent questions about health systems. It aims to strengthen the quality and efficiency of health policy-making.^15,16^ The unit is staffed by two to three people, and service users are mainly central and local public sector policy-makers.

The future of this knowledge translation platform is, however, in doubt, as has often been the case with projects funded by donors and grants. The transition of such units into local ownership and ongoing sustainability (or ‘institutionalization’) has often been fragile. Many have collapsed at the end of the donor funding period.^17,18^ The purpose of this study was therefore to describe the process and extent of institutionalization of the health policy rapid response unit. It also aimed to provide advice about how the process could be supported and improved in the future, to inform the future development of both this and other units.

### Institutionalization

Institutionalization is the process by which a set of activities becomes an integral and sustainable part of a formal system. It can be seen as a sequence of events leading to “new practices becoming standard practice.”^19^ In this study, institutionalization refers to the continuation of the rapid response unit after withdrawal of support from the SURE project funded by the EU.

### Study Framework

Berger and Luckmann^20^ noted that “habituation precedes any institution.” Institutions whose activities were based primarily on recurrence were found to be considered “natural,” predictable and stable.^21^ The World Bank produced a three-part framework on institutionalization, covering:

(i) Existence of an institutional framework;
(ii) Consistent production of relevant reports;
(iii) Adequate resource availability.

The first part of the framework suggests that habituation can be linked to the existence of an institutional framework (with a favorable environment and governance structure) that will encourage the ongoing performance of the activities concerned.^22^ This includes a formal mandate for the existence of a unit to implement those activities.^23^ This mandate clearly defines the roles and responsibilities required to avoid duplication of effort, maximize productivity and enhance understanding of stakeholders’ needs. As a result, a work plan and procedures are generally respected by all actors. The mandate also allows the development and consolidation of the habits of action within the organization.

Once a formal mandate has been provided to a unit, its activities can become normal behavior and be widely accepted. The activity is then not specific to the context or people involved but has been institutionalized.^24^ Standardized behavior includes setting up consistent and routine data collection, data analysis and reporting.^19,22^

The production of consistent data and the dissemination of results play a crucial supporting role in the institutionalization process. The World Bank framework noted that consistent data production develops capacity for consistent estimation methods.^22^ Information production and communication therefore facilitate implementation, and also encourage advocacy, strengthen the recognition of the unit’s merits, and support routine reporting. Routine data production needs to become a habit, but it takes time for this to happen.^25^ Individuals are affected not only by empirical findings, but also external realities which are imposed on them.^26^ This applies both to the production of relevant reports and their use in decision-making and policy processes. This is therefore an important measure of the level of institutionalization.

One of the last steps in institutionalization is the availability of enough resources to implement activities.^22^ Institutionalization requires institutional learning and knowledge transfer within the unit.^27^ The transfer of knowledge about activities between generations of members of the unit assumes the presence of well-trained and capable human resources. This also implies the availability of financial and material resources to support continuity. The continued implementation of the activities helps to embed them as routines.^27^ The routine first becomes the norm, then gradually becomes a standard practice that is sustainable over time.

Complementing the World Bank framework, five main transitional phases have been suggested for institutionalization: awareness, experimentation, expansion, consolidation and maturity.^28,29^ Each phase has particular characteristics and strategies. We used the World Bank three-part framework to measure the institutionalization of Burkina Faso’s rapid response service. This framework has been previously used to evaluate and assess institutionalization processes. Its use therefore ensures that this study is comparable with others. We grouped the five institutionalization phases under each of the three institutionalization factors in the World Bank framework to provide a structure for data analysis.

## Methods

This research was a qualitative and exploratory case study of the process and extent of the institutionalization of the Burkina Faso rapid response service. The study period was from March 2011 to August 2015 and included pilot and scale-up phases of the rapid response service implementation in Burkina Faso.

### Data Sources and Recruitment

Data were collected from interviews, documentation covering the rapid response unit’s implementation and strategies, and the personal knowledge of the principal researcher.

Documents from the unit were used to identify information about policy-makers using the service (for example, their profiles, locations, questions asked and how frequently they sent questions and used the service). These documents also supplied information about the unit’s infrastructure (for example, its equipment, internet access and budget), funding and support (for example, the mandate, government willingness to support the unit and supplement EU funding, and activities supported by EU funding). Finally, the documents also recorded whether service users felt that the briefs produced by the unit were useful.

Potential interviewees were identified depending on how often they used the service and how aware they were of the unit’s work. Emails and phone calls were used to make contact before the interview. In total, 18 people were interviewed for this study: all were policy-makers within the Ministry of Health. An outline of the interview guide is given at Appendix 1. Each interview was audio recorded, and notes were also taken by the interviewer. All but one of the participants were interviewed face-to-face; the other was interviewed via Skype, while out of the country.

The principal investigator acted as a participant observer, having been the first full-time member of staff in the rapid response unit, and responsible for its implementation since January 2011. This researcher documented the implementation of the rapid response unit and service, including strategies, budgets and policy discussions. The study therefore drew on this researcher’s extensive knowledge of each phase of the service implementation to add details to supplement other resources. In particular, the researcher supplied more information about the type of policy-makers using the service. This included their location, the frequency of requests for briefs, and the types of questions asked. He also supplied documents about the progress of implementation of the unit, including development of its formal mandate and access to resources, and budget provision.

To ensure that the study remained objective, two other researchers were periodically consulted to triangulate the data and assure completeness of methods and findings. These researchers are co-authors of this paper, and were both working in the Ministry of Health, responsible for implementing the SURE project.

### Data Analysis

The data obtained from interviews were transcribed and coded by hand.^30,31^ The interviews were analyzed using qualitative thematic analysis.^25^ The relevant documents and reports were systematically tabulated and analyzed. Data from the document analysis were triangulated with the qualitative data from interviews.^32,33^

## Results

Table 1 shows strategies used in the rapid response unit across each of the main elements of the World Bank Framework, and the five phases for a service institutionalization process.^17,26^ These are the internal elements around which the unit concentrated its efforts to ensure its long-term survival: its ‘action plan’ for institutionalization.

**Table 1:** Characteristics and Strategies of Each Stage of Institutionalization^22,29^

**Phases**	**Existence of an Institutional Framework**	**Consistent Production of Relevant Reports**	**Adequate Resources**
	**Strategies**	**Strategies**	**Strategies**
Awareness	Demonstrate the need for improvement in the policy process.	Discuss the format of rapid response briefs.	Demonstrate the need for improvement in the policy process.
	Create awareness of the new service among policy-makers.	Create awareness of the format of briefs among policy-makers.	Demonstrate the need for resources to implement the rapid response service.
	Plant the idea of evidence driving improvement.	Plant the idea of evidence driving improvement.	Plant the idea of evidence driving improvement.
Experimentation	Implement small-scale activities to test the new service and demonstrate need for institutional support.	Implement small-scale activities to test the new service.	Implement small-scale activities to test the new service.
	Develop work plan, use guide materials, and discuss how the unit fits with existing units.	Learn from elsewhere, and from meetings and workshops.	Develop budget plan and document needs (financial, equipment, internet connection).
	Discuss the government mandate.	Recruit policy-makers and start to get requests for briefs.	Discuss the location of the unit with stakeholders.
		Share examples of briefs from elsewhere and develop our own.	
Expansion	Develop expansion strategies (goals, priorities, implementation plans).	Develop expansion strategies (goals, priorities, implementation plans).	Develop expansion strategies (goals, priorities, implementation plans).
	Develop consensus among policy-makers that the service should be continued.	Increase organizational capacity to support the unit.	Build capacity and develop leadership in the unit.
	Share innovation, cost savings and results.	Strategic expansion of unit activities in scale and scope.	Share innovation, cost savings and results.
	Demonstrate improved service quality through the unit’s activities.	Demonstrate improved service quality through the unit’s activities.	
Consolidation	Identify missing or lagging activities and providing them.	Embed unit activities into standard organizational operations, and add lagging or missing activities.	Recruit and train additional staff.
	Enhance coordination of unit strategy and activities.	Fully integrate a balanced set of activities into the everyday working of the Ministry.	Develop mechanism to motivate staff.
		Continue support for learning within and beyond the unit.	Obtain state budget line for the unit.
Maturity	Values, leadership, policy and resources are sufficient.	Unit is carrying out all possible activities.	Office, equipment, and high speed internet is available to the unit.
	The service is formally and philosophically an integral part of the health system.	Unit has access to suitable evidence databases.	Values, leadership, policy and resources are sufficient.
		Unit is sharing experience with other countries.	State budget line is available for the unit.

Table 2 summarizes the principal findings from the data collection. It shows the location of each element of the rapid response service and the phase of its institutionalization: the reality of the progress of institutionalization over time. It helps to identify where effort should be focused to achieve full institutionalization of the rapid response unit.

**Table 2:** Evolution of Essential Elements of the Rapid Response Service During Each Phase of Institutionalization

**Institutionalization Phase**	**Institutionalization Elements**		
	**Existence of an Institutional Framework**	**Consistent Production of Relevant Reports**	**Adequate Resources**
Awareness	• Awareness phase started from October 2010 to February 2011 • At the beginning of the SURE project, key policy actors were introduced to the unit concept by the project leaders • SURE project leaders used meetings to emphasize the potential value added by the unit • Key decision-makers became aware of the unit and were willing to allow its establishment even without formal approval • Key high level policy-makers approved the formation of the unit as a pilot • Key leaders agreed to support the unit • Key decision-makers became aware of the benefits of the unit and agreed to create mechanisms to strengthen it	• Key stakeholders expressed a need for unit briefs for urgent decision-making • Unit leaders were encouraged to explain the new service during official meetings • No Burkina Faso briefs were available, but a Ugandan brief was used as an example	• Minimum resources made available through donor support (SURE project money) for the pilot • Officials provided support for further examination of options for improving policy-making • Staff drew on experience from elsewhere, including other countries with similar units • Unit leaders recognized the need to develop mechanisms or processes to reward quality work and staff effort • The EU supported the rapid response unit with 16 500 USD, for capacity building, for experience learning elsewhere for one person, meetings, advocacy, and communication • It was agreed that staff needed to speak English • The issue of internet access was raised
Experimentation/pilot	• Experimental phase started from March to December 2011, without formal policy approval • Consensus developed among stakeholders of the importance of including the unit in the policy process • There was a discussion about including the unit in wider Ministry of Health reform process • The power to extend the unit’s activities was delegated to the Research Directorate for Health, and the unit was given access to directorate resources • Fundamental values supporting a culture of quality were set out and accepted by unit staff • The Ministry of Health officially allowed unit staff to contact policy-makers directly	• The unit was provided with management, facilitation and coaching and access to the required knowledge and skills • A consensus developed on how to generate capacity to enable experimentation • Briefs from Uganda were used as examples • Policy-makers using the unit documented activities and shared this with others • Implementation guidelines and materials were developed • The unit was working with a small network of five to ten policy-makers • The unit produced at least two briefs per month, continuing to draw on advice from the Ugandan unit to improve their quality	• EU funding was used to finance the experimentation costs, and resources were made available to visit the Ugandan unit • Office, internet, and equipment were provided by the Research Directorate for Health • Unit leaders recognized the need for supervision, coordination and clear responsibilities for unit expansion, which were provided by SURE project leaders • A consensus developed about appropriate and effective structures for the pilot phase • Managers accepted that resource constraints would affect quality, and so developed incentive programs to improve quality
Expansion	• Expansion phase ran from January 2012 to August 2015 (44 months), and involved recruiting more policy-makers, 23 in total • In 2012, the unit was officially included in the Ministry’s organization chart, and incorporated into the directorate’s strategic documents • Expansion policies were developed (implementation guidelines, tools, flyers, and other communication materials) • Policies were developed to encourage unit evaluation and continuous improvement • The staff were able to articulate the fundamental values of the unit • The unit’s core values could be seen to guide its development	There was a critical mass of competent staff to support the unit’s work • The unit’s manager led by example, to demonstrate the unit’s values and procedures • Mechanisms and systems were established for routine documentation of results and to support learning • The unit’s leaders were involved in ongoing advocacy for the service • The unit began to work with more policy-makers, including at the subnational level, and produce more briefs • 23 Policy-makers sent 78 questions in 44 months, with an average of 1.7 questions per month	• Realistic unit budgets were established, based on the true costs of implementation • A dedicated staffing level of three was agreed • New staff were hired, but retention was a problem because of working conditions (language and internet problems) • A directorate was identified to house the unit, and resources provided, but internet access remained a problem • Unit strategies were refined after internal evaluation • The Ministry of Health agreed to reward quality but did not provide additional funding • The need for a budget line was raised but not resolved, although EU funding was made available to support ongoing work • The EU supported the expansion phase with US$23 616 for salaries for two people, advocacy activities, IT equipment maintenance and internet connection office supplies, and travel
Consolidation	• Unit policies for continuous improvement were established. Resources were available and core values and policies are consistent • Operational managers feel responsible for the quality; they provide leadership for the unit’s activities and are willing to promote it • The performance of staff and managers reflects the unit’s fundamental values • Awards and programs are in place to support these values • Since June 2013, changes within the unit’s home directorate have led to some instability within and beyond the unit	There is a mechanism to perpetuate the expertise of the unit, and systems for documentation, information-sharing and advocacy operate routinely • Some policy-makers routinely send questions but others need to be encouraged • The unit provides evidence for policy development and other work • Briefs are used for decision-making but tracking this is not always easy • The unit has been asked to look at the use of evidence in policy-making, which is beyond its mandate	• Estimated resource needs are incorporated into the annual strategic plan but not funded The unit has additional staff supporting work on briefs • All unit staff can describe how their work contributes to improving quality of briefs for urgent decision-making • The Ministry has committed to providing resources, but not yet made them available • State budget paid salaries, office and equipment amounting to US$6345 • The staff believe reward mechanisms are fair • There are mechanisms to enable policy-makers to suggest how to improve the unit’s work • The unit has an office, but internet capacity is still limited and staff cannot download evidence on site • Advocacy for a government budget line continues after SURE project funding ended

Abbreviations: SURE‏, Supporting the Use of Research Evidence; EU, European Union.

The next sections draw from the interview data to explore each of the three areas of the World Bank framework in more detail.^22^ They examine the implementation process, and assess which stage^29^ the unit has reached for each area.

### Existence of an institutional framework

Government practice in Burkina Faso’s health system is that service implementation requires several elements. These include approval from high-level policy-makers, the establishment of technical committees and a decree signed by the Minister of Health or the Secretary General of the Ministry of Health. This process was started for the rapid response unit, and meant that many policy-makers were aware of its existence before implementation. One of the SURE project implementers said:

*“…at the earlier stage of SURE project activities implementation, including the Rapid Response Service, we presented the project proposal at a committee meeting chaired by the secretary general of the Ministry of Health. As you know, we also introduced the Rapid Response Service mechanism during the EVIPNet annual meeting held in 2010 and participants were excited to see the Rapid Response Service briefs and see how it would help them in their policy making..*.” (Senior policy-maker).

The pilot phase, however, which corresponds to the experimental phase of the study framework,^29^ began before this policy process was complete. This was because of the involvement of the Minister of Health’s adviser. He was one of the key players responsible for the SURE project in the country, which supported the rapid response service. He and the principal researcher, in his role within the unit, therefore took action to raise awareness and attract business by direct personal contact and presentations about the use of the rapid response service. The adviser also started to suggest that the unit should be incorporated formally into the Ministry of Health’s organization chart.

During the pilot phase, policy-makers began to familiarize themselves with the rapid response service, showing that the use of “simple rules” is acceptable without a formal mandate such as a decree.^34^ One interviewee noted that providing value was probably more important than formal mandate for policy-makers to use the service, but the formal mandate was required for long-term survival:

“…*before policy-makers support service implementation, you should show the added value of the service and after that, you will see every policy-maker defending the service at decision-making meetings. Usually, to sustain service implementation within the Ministry of Health, [you need to] make sure you get an official mandate from the Minister of Health…”* (Senior policy-maker).

As an organization progresses towards the expansion phase, more specific operational policies become important.^35^ As the scale and scope of the rapid response unit’s activities widened, the principal researcher, as unit head, observed that more clarity was needed on what could be delivered to particular deadlines, especially because of problems with internet access. It would also have helped to have flexibility in the implementation and the delegation of powers to carry out improvements. During this phase, the unit recruited more policy-makers at national and sub-national level, and developed written guidelines for the service. Feedback from service users was positive. The project also received its official mandate in this phase. In 2012, the Ministry of Health officially included the service in its organization chart, as part of the general directorate for health information and health statistics (DGISS).^36^ This gave the unit access to additional directorate resources for implementation. It will eventually enable the rapid response service to obtain a budget line in the Ministry’s state budget. One policy-maker noted:

“….*because the rapid response service is new in the health system, maybe to make sure you get permanent financial resources, it should be located under another unit, under an influential director who will able to defend the service anytime…..also during the unit’s period of planned operational donor funding, the rapid response service can present its operational plan and get funded by the donors”* (Senior policy-maker).

### Consistent Production of Policy-Relevant Reports

The consistent production of policy-relevant reports was crucial to the unit’s survival. Policy-makers were quick to see the value of the unit’s reports, with one saying:

*“…In one of the policy meetings, one of your briefs was important to me; I was speaking with evidence, papers, references and examples from other countries. When I started to talk, the others could not speak again because I was not talking about my experience but evidence…some of the summaries in your brief were part of our meeting conclusion”* (Senior policy-maker).

Another service user also noted, however, that maintaining quality was vital:

“…*for example, if I have a meeting or a discussion with my supervisors and if I send you a question, and you cannot send me a brief with substantial evidence to support my arguments by the time that I need it, do you think I will come back to you again?”* (Junior policy-maker).

Information and communication between the unit and policy-makers were therefore important to ensure that the right questions were answered in the right time-frame. They also strengthened awareness and recognition of the merits of the service. Each phase of institutionalization presented different challenges. These included

- The definition of specific goals for data collection and communication;
- The identification of obstacles to the achievement of these goals;
- The development of profiles for the different target audiences (small or larger policy groups at the central or sub-national level, within or beyond the Ministry of Health);
- The segmentation and prioritization of target audiences;
- The choice of communication messages, equipment and channels for each group;
- The pre-testing of service outputs;
- The evaluation of user satisfaction and the use of feedback to refine communications; and
- Recognizing and rewarding staff.

Sharing practices from Uganda^13,14^ was useful to increase stakeholder interest during the awareness phase, which was in turn useful for the pilot phase. For example, one of the first users of the service observed:

“*My decision to use the service came after you showed me an example [brief] from another country [Uganda].…although it was in English, the format was really attractive and user friendly, and I was able to read it and understand the content….Once you sent back the briefs, I and another user, I believe, would also have loved to get the paper from where you summarized your responses*” (Policy-maker).

There were clear indications that the unit was moving into the next phase. These included regular documentation from those using or experimenting with the rapid response service, and the sharing of experience with various target audiences. During the experimental phase (from March to December 2011), the unit worked closely with Uganda’s rapid response service to deliver five briefs, under the supervision of two of the researchers, who had responsibility for implementing the SURE project in Burkina Faso. This gave the team the opportunity to learn from the Ugandan experience. The experimental phase started with four policy-makers from the central level (based in the capital city, Ouagadougou). Five briefs were delivered in 9 months.

Delivery during the awareness and experimental phases increased expectations among stakeholders that the unit would be able to deliver high-quality briefs on demand. As demand increased, this became difficult to manage because of the unit’s limited resources. Of the 64 questions that fit the remit, the unit was able to respond in a timely way to just 51, or 79.7%. The main reason for not being able to address the other questions was often the poor internet connection in the Ministry of Health, or in Ouagadougou. Other reasons included not being able to find evidence to address the question on time, or cancellation of the need from the user, for example, because their policy meeting was cancelled. Some questions were sent late, giving only a day to respond. This caused some problems, as one participant from the Ministry of Health commented:

“…*at the beginning I was getting my briefs on time from the rapid response service and they were relevant, but I did not get my last brief on time. We understand that you sometimes have difficulties, but we do not want to get used to something that you cannot continue...”* (Senior policy-maker).

It is fair to say that some of the briefs did not meet the users’ needs for speed, quality and contextualization. The unit almost became a victim of its own success, and can reasonably be said to remain at the expansion phase for this criterion. Improving the quality of delivery with increased resources may be essential to move beyond this. Currently, the unit does not track the use of its briefs to ensure that they inform policy-making, and this evaluation will be necessary to move to the next phase.

### Adequate Resources

The resource requirements for the rapid response service varied across the phases of institutionalization, but various investments have been recognized as necessary to enable the unit to reach maturity (for example, capacity building, experimentation, learning English, and the time dedicated by officials to supporting the process of institutionalization). The size of these investments varied over time.

At the awareness phase, some high-level decision-makers saw the service as too expensive. However, the unit was able to demonstrate that activities like elimination of errors, duplication and inefficiencies in service implementation would save money. The unit start-up required some additional resources, but the actual costs related to the unit’s operation depended on staff time. During this phase, resources were needed for salaries, capacity building, to learn from experience elsewhere, meetings, advocacy, and communication by the SURE project team. These costs, estimated at US$16 500, were fully funded by the EU. Of these costs, 47% was for capacity building and learning from Uganda, 38% for equipment and 15% for advocacy activities at the central level.

The resources for the experimentation phase (US$13 095) were also provided by the EU and covered salary (56%), advocacy meetings (10%), guideline development (10%), internet connections (7%), in-country training (7%) and office supplies (1%). The total cost for the expansion phase was US$23 616 and was also fully funded by the EU. During this phase, the Ministry of Health held some discussions about supporting the unit through provision of an office, additional staff, and equipment. The expansion cost covered salaries for two people (79%), advocacy activities (10%), IT equipment maintenance and internet connection (8%), office supplies (2%), and travel (1%). The resource needs during the expansion phase included capacity-building, communication mechanisms, reward systems and leadership development. Many of these costs required initial investment but were expected to decrease later. During this phase, the unit was allowed to draw on Ministry resources, although its main source of funding remained the EU.

Finally, the unit has now moved into a transition period in terms of resource availability, which probably corresponds to the consolidation stage in our framework.^29^ The budget for staff salaries, office and equipment (US$6345) was funded by the government. The office, however, had no internet connection, and there were no funds to support additional activities such as communication, advocacy, and training. There was also still no dedicated budget line for the unit in the Ministry of Health’s accounts. This was often mentioned by study participants, with one policy-maker suggesting:

“…*in the Ministry of Health, to guarantee resource availability, you should make sure there is a budget line code for the rapid response service in the state budget*” (Senior policy-maker).

## Discussion

Data from interviews, documents and the knowledge of the principal researcher demonstrate that the rapid response unit has moved towards institutionalization, and is becoming a formal part of the health policy system.^19^ We consider that the unit will be fully institutionalized, or ‘at maturity’ when it is part of the Ministry of Health’s organization chart, with a clear mandate and dedicated budget line, and when a majority of policy-makers regularly send requests for information, and use the briefs supplied to support decision-making.

Our results suggest that the unit is further ahead on some areas than others. First, the mandate for the unit was clearly established during the pilot phase. The unit was included within a directorate at the Ministry of Health shortly after the pilot was completed, during what we have characterized as the expansion phase.^36^ This would suggest that the unit is reasonably well-established in formal terms, with a clear understanding available of its place in both the structure and function of the Ministry. We suggest that in terms of a clear mandate, the unit is well into the consolidation phase, and could even be considered as reaching maturity.

Second, influential decision-makers were aware from the start that the unit could make a valuable contribution to policy-making.^13,37^ In particular, detailed briefs were seen to save time and cost and increase visibility in decision-making.^38^ Policy-makers commented that the briefs enabled them to speak with authority in meetings. The expansion of operations from ‘a few’ to ‘the majority’ of policy-makers is a process that will take time. However, the unit has seen its operations expand in both scope and scale during its lifetime. Expansion has been geographical (from central to regional level), reinforced by increasing the number of policy-makers using the service and therefore the number of questions received. SURE project guidelines, the pilot phase in Burkina Faso and the experience in Uganda all suggest that the best strategy is to start with a small group and keep recruiting among the same target group.^13,39^ In this area, therefore, the unit is probably at the expansion to consolidation stage.

However, the issue of resources has been harder to address. Most of the financial and technical support for the unit has come from the SURE project via EU funding. Units that are entirely dependent on donor resources have tended to close or end at least some of their activities when the external funding stops.^17,18^ While some government funds have been provided in recent stages, this funding falls far short of what has previously been provided by the EU. Adequate internet access remains a challenge, although it is vital for the unit’s ongoing function. If the government is serious about retaining the unit, it will need to provide dedicated funding and adequate resources. Without these resources, the unit will fail despite its clear mandate, because it will be unable to satisfy the needs of policy-makers, who will therefore cease to use it.

Both Tables 1 and 2 show a theoretical state, with clear separation between the phases of institutionalization. However, it is clear from Table 1 that there is potential for the strategies to overlap between phases, and from Table 2 that the unit moved forward at different speeds across different areas. In practice, therefore, this study shows that the phases of institutionalization^29^ overlap, and that there is often little clear demarcation between them.

It was reasonably easy to identify the end of the pilot (experimental) phase and the beginning of the expansion phase. However, it was much harder to tell where exactly the expansion phase ended, and the consolidation phase began. This confirms that in practice, there is considerable overlap between phases. This is partly because of the challenges to implementing a rapid response service in a low-income country.^40,41^ First, recruiting policy-makers to send questions was difficult. Second, when they started to use the service, it was hard to support and retain them, while also recruiting additional policy-makers. The unit struggled to maintain the quality and speed of response as demand grew. In practice, it proved impossible to satisfy all the policy-makers, and some did not send any further requests. Third, internet availability and access to evidence to provide timely responses to urgent requests was an issue. When expansion strategies are implemented, they bring forward or encourage the need to make an assessment of activities. This paves the way for a review period, involving professional development, adjustment, and coordination.^42^ This naturally leads to consolidation. At the same time, however, expansion was still ongoing, with both awareness-raising activities and additional recruitment of policy-makers.

Previous work^12^ suggested that linking research to action required activity across a range of areas. The general climate must support the link between research and action. We have seen in the rapid response unit that backing from a senior figure (the adviser to the Minister for Health) was an essential driver in starting to implement the unit. There also needs to be both ‘user pull’ and ‘push’ to disseminate research findings. The unit’s progress shows that while policy-makers needed to provide questions (‘user pull’), the unit staff and advocates for its success also needed to spend time raising awareness and encouraging policy-makers to consider using the service.

The unit’s mandate does not cover other elements of the framework,^12^ such as influencing the production of research, or linking policy-makers direct to researchers. Senior decision-makers may need to consider whether these activities are also necessary to increase the use of evidence in policy-making. If so, the unit’s mandate might be expanded to cover them, or another unit given this task. Future studies may help to address this point. The findings of this study also show that the unit’s activities have, to date, not addressed the final component of the framework, evaluation. The unit head kept records of whether the briefs were considered useful by policy-makers. It may, however, be helpful for the unit to track the effect of its briefs on policy. This would enable it to assess both that the briefs informed policy-making, and also whether policy-making supported by evidence is genuinely more effective. This would enable the unit to ensure that its continued existence and institutionalization can be justified.

This study of the institutionalization of the rapid response unit is the first of its kind in Burkina Faso. However, at the international level, the World Bank has conducted case studies in eight countries to examine the specific challenges of institutionalization.^43^ The lessons learned from other countries on health units’ institutionalization suggest that weaknesses in data-generating systems and links to public expenditure management (especially in a decentralized context), together with difficulties in tracking private health expenditure flows, are among the main reasons why progress toward institutionalization has been minimal.^43^ This suggests that this unit is not unique in struggling with resources to generate quality reports, and also that evaluation of its impact will also be key to full implementation.

## Strengths and Weaknesses of the Study

The principal investigator had a key role as a participant-observer in the rapid response service implementation process. This could be both a strength and a weakness. The involvement of a researcher provides a check against participants’ subjective reporting, and helps to ensure consistency, along with other checks and balances. Participant observation is valuable for understanding the physical, economic, cultural, and social contexts in which interviewees live and work. It also informs understanding of the relationships between stakeholders, ideas, contexts, norms, events and behaviors and activities related to unit institutionalization.^44,45^ It can, however, introduce bias, particularly in selection of data supporting certain points of view. In this study, we therefore took steps to avoid bias. In particular, we engaged external experts who were familiar with the rapid response service and the health system in Burkina Faso to review the study’s major findings.

The second strength is that the study drew extensively on work from Uganda, as well as the World Bank framework and other literature, to understand the process required to establish a rapid response service unit in Burkina Faso, and particularly to identify factors that could affect institutionalization in Burkina Faso.

This study also had some weaknesses. The first is that it drew on policy-makers as interviewees. Their knowledge of the service, however, was limited to using it, and many did not know much about its early organization. These data were supplemented by the knowledge of the principal researcher, who had been involved in the unit from its inception. The sample size was also relatively small, at just 18 interviewees. This is, however, reasonable for a qualitative study, particularly because the available population (those who had used and understood the service) was also limited.

## Implications for Future Studies

The study has evaluated the process and extent of the institutionalization of the health policy rapid response unit in Burkina Faso. It has focused in particular on the earlier stages of the unit’s implementation, when the unit was mainly funded by the EU. This touches on one of the most intriguing areas for further research about the sustainability and long-term financing of such units. We therefore suggest that future studies might investigate the funding mechanism of the unit more closely. In particular, they might evaluate the level of national ownership needed to support such units in the longer term. Researchers might also explore the benefits of the unit’s activities for urgent decision-making and the use of evidence to support policy-making.

This study used five main transitional phases to examine the institutionalization of a particular unit. It therefore contributes to our understanding of the dynamics linking institutionalization to each of these five phases. However, it has also highlighted some gaps in the activities of the unit. Future studies should evaluate, for example, gaps in the service. They could also explore the expansion and consolidation phases, examine the impact of the unit and look at resource availability. All these affect the long-term future of the service.

In the section on consistent production of policy-relevant reports, we pointed out that tracking the use of the briefs for urgent decision-making is not always easy. Demand from policy-makers has to be stimulated by personal contact to generate and encourage new users. More investigation of the impact of the rapid response unit’s briefs in the policy-making process would be a useful area to explore, as would how the service attracts new users.

## Implications for Policy and Practice

This study reports on the implementation of a small unit providing a rapid response evidence synthesis service in Burkina Faso. In practice, the different steps highlighted in unit institutionalization could be used to support and strengthen the implementation of other units in Burkina Faso, or rapid response services in other countries. The study will be useful to policy-makers in considering specific phases and the factors that are the most relevant to each. In Burkina Faso, the study framework and findings could assist the Ministry of Health to plan and focus its efforts and resources to strengthen and sustain the unit’s implementation.

## Conclusion

The institutionalization process for the rapid response service in Burkina Faso has been fluid rather than linear. There were several key components that led to service institutionalization. First, the unit obtained an official government mandate. Second, it proved that it could produce briefs in response to urgent questions from policy-makers in a timely manner, providing appraised and synthesized research evidence. Finally, the unit needs permanent availability of financial resources. Five main transitional phases have been used to assess service institutionalization. These were awareness, experimentation, expansion, consolidation and maturity. The service has largely reached the stage of either expansion or consolidation, but will require considerable additional effort to reach maturity.

## Acknowledgements

The authors thank the study respondents who willingly gave up their time to participate in the interviews and provide feedback on the manuscript. This work was supported by the International Development Research Centre (IDRC) International Research Chair in Evidence-Informed Health Policies and Systems (providing funding for Nelson K. Sewankambo, Makerere University, Kampala, Uganda and John N. Lavis, McMaster University, Hamilton, ON, Canada) in partnership with the SURE for policy in African health systems project in Burkina Faso, funded by the EU.

## Ethical issues

This work was approved by the School of Medicine Research and Ethics Committee of the College of Health Sciences, Makerere University, Kampala, Uganda and the Burkina Faso Ministry of Health Ethical Committee. The objectives of the study were explained to participants and informed consent was obtained from all participants.

## Competing interests

Authors declare that they have no competing interests.

## Authors’ contributions

AZ developed the methodology, collected the data, performed the analysis, and wrote the manuscript. JNL and NKS were involved in the conception, design and implementation of the study and in drafting the manuscript. BK and SO were involved in the conception of the study and drafting the manuscript. All authors read drafts of the manuscript and approved the final version.

## Authors’ affiliations

^1^Clinical Epidemiology and Biostatistics Unit, College of Health Sciences, Makerere University, Kampala, Uganda. ^2^Ministry of Health, Ouagadougou, Burkina Faso. ^3^McMaster Health Forum, Centre for Health Economics and Policy Analysis, McMaster University, Hamilton, ON, Canada. ^4^Department of Health Research Methods, Evidence and Impact, and Department of Political Science, McMaster University, Hamilton, ON, Canada.

## Key messages

**Implications for policy makers**

- The rapid response service has emerged as a streamlined approach to supporting urgent decision-making by policy-makers, enabling evidence to be synthesized in a timely manner.
- Institutionalization of a service like this requires a stable environment, a mandate from the government, and sufficient financial and human resources.
- Having a state budget line for a service will guarantee resource availability.
- The institutionalization of a service like this does not necessarily follow a linear process, and those responsible should be prepared for this.
- Even after a service has reached the consolidation stage, additional efforts may be required to help it reach maturity.

**Implications for public**

The rapid response service is a small unit within Burkina Faso’s health ministry, synthesizing evidence in a timely manner to support urgent decision-making by policy-makers. It is hoped that this will result in policies that are informed by evidence, and therefore more likely to be cost-effective, and not waste taxpayers’ money. A more efficient policy-making process (with less time spent debating or trying things that are not supported by evidence) is also better value for taxpayers. This paper explains the process of establishing and building up such a unit, and how this can be supported to make it more likely to succeed. It is hoped that it will therefore be useful in supporting the setting up of future units in other policy areas or elsewhere in the world. This should enable a more rapid return on investment and secure foundation for these units.

## Appendix 1

Appendix 1. Outline of Interview Questions The interviews covered three areas.
**1) The rapid response service implementation environment**

What are the environmental factors which could lead to the institutionalization of the rapid response service?

What are the political factors which are important to the ongoing success of the unit?

Is there legislation or a policy that was required for the unit?

Should the unit be incorporated into an existing directorate or should it exist as an independent entity? If yes which directorate do

you think should be good for the rapid response service?

How can the service be institutionalized? Based on your experience in the Burkina Faso health system what are the different steps to institutionalized a unit like the rapid response service?

**2) Production of consistent data and dissemination of results**

What do you see as the mechanisms of accountability within the unit?

How should data be presented to support the unit’s institutionalization?

How must the data be collected, stored and analyzed?

What should be the frequency of data/rapid response briefs production?

Must there be a strategy for dissemination of results? How can we track the use of the rapid response brief for decision-making?

Who might be the main actors to use the rapid response service? And how can we recruit them and maintain them to frequently use the rapid response service for their urgent decision-making?

Should the data and results be used for the development of policies or to support decisions?

**3) What do you see as the human, equipment and financial resources needed by the unit in the longer term (both quality and quantity)?**

What does the unit’s capacity need to be to do its job?

What are the hardware resources needed by the unit (quality, quantity, type)?

We have challenges maintaining coworker in the rapid response service because of the language issue to summarize the briefs (most of the evidence are in English) in the Francophone country, what is your advice?

Internet connection is one of the main resources de deliver the briefs on time, what might be the unit strategy to get permanent/ stable internet connection?

How can the unit be funded in the longer term? What funding sources must it have?

State budget line is one of the key components to support the rapid response unit financial institutionalization, how that can be done? What is the usual process for a unit in the Ministry of Health to be funded by the state budget?
